# Retraction Note: Synergistic renoprotective effects of sesame oil and erythropoietin on ischemic kidney injury after renal transplantation

**DOI:** 10.1186/s13568-026-02035-3

**Published:** 2026-03-06

**Authors:** Liyu Ye, Fang Xiao, Jijun Xie, Lingling Feng, Zhangjun Tang, E. Chen, Chuchu Chen, Bowen Xu, Ronghai Deng

**Affiliations:** Second District of Organ Transplant, First Affiliated Hospital of Sun Yat-Sen University, Guangzhou, 510080 China

**Retraction note to: AMB Expr (2020) 10:4** 10.1186/s13568-019-0934-y

The Editor-in-Chief has retracted this article because it contains data that overlaps with data from the following article, (Wang et al. 2020), also published by AMB Express. In addition, an investigation conducted after the publication determined that the two articles were under consideration by the journal at the same time. The authors did not provide an explanation for this data overlap. The Editor-in-Chief therefore no longer has confidence in the results and conclusions presented in this article.

The authors did not reply to correspondence from the Publisher about this retraction.

## References

[CR1] Wang Z, Sun W, Sun X et al (2020) Kaempferol ameliorates cisplatin induced nephrotoxicity by modulating oxidative stress, inflammation and apoptosis via ERK and NF-κB pathways. AMB Express 10:58. 10.1186/s13568-020-00993-w 32219583 10.1186/s13568-020-00993-w PMC7098399

